# Supplementary material for: Application of clown care in hospitalized children: A scoping review
Source: PLoS One. 2024 Dec 19;19(12):e0313841. doi: 10.1371/journal.pone.0313841 (PMC11658477; doi:10.1371/journal.pone.0313841)
Supplement: S2 File — (DOCX) [file pone.0313841.s002.docx]

**S2 File.**. **Search strategy.**

Take the PubMed database retrieval strategy as an example

| Date |  | Keywords | Search results | |
| --- | --- | --- | --- | --- |
| 10/12/2023 | #1Clown [Title/Abstract] OR medical clown [Title/Abstract] OR clown doctor [Title/Abstract] OR clown intervention [Title/Abstract] OR clown therapy [Title/Abstract] OR clown nurse [Title/Abstract]  #2child [Mesh Terms] OR children [Title/Abstract] OR pediatric [Title/Abstract] OR kids [Title/Abstract]  #3 #1AND#2 | | 1082 |  |
