# Supplementary figures and images for: Application of clown care in hospitalized children: A scoping review
Source: PLoS One. 2024 Dec 19;19(12):e0313841. doi: 10.1371/journal.pone.0313841 (PMC11658477; doi:10.1371/journal.pone.0313841)

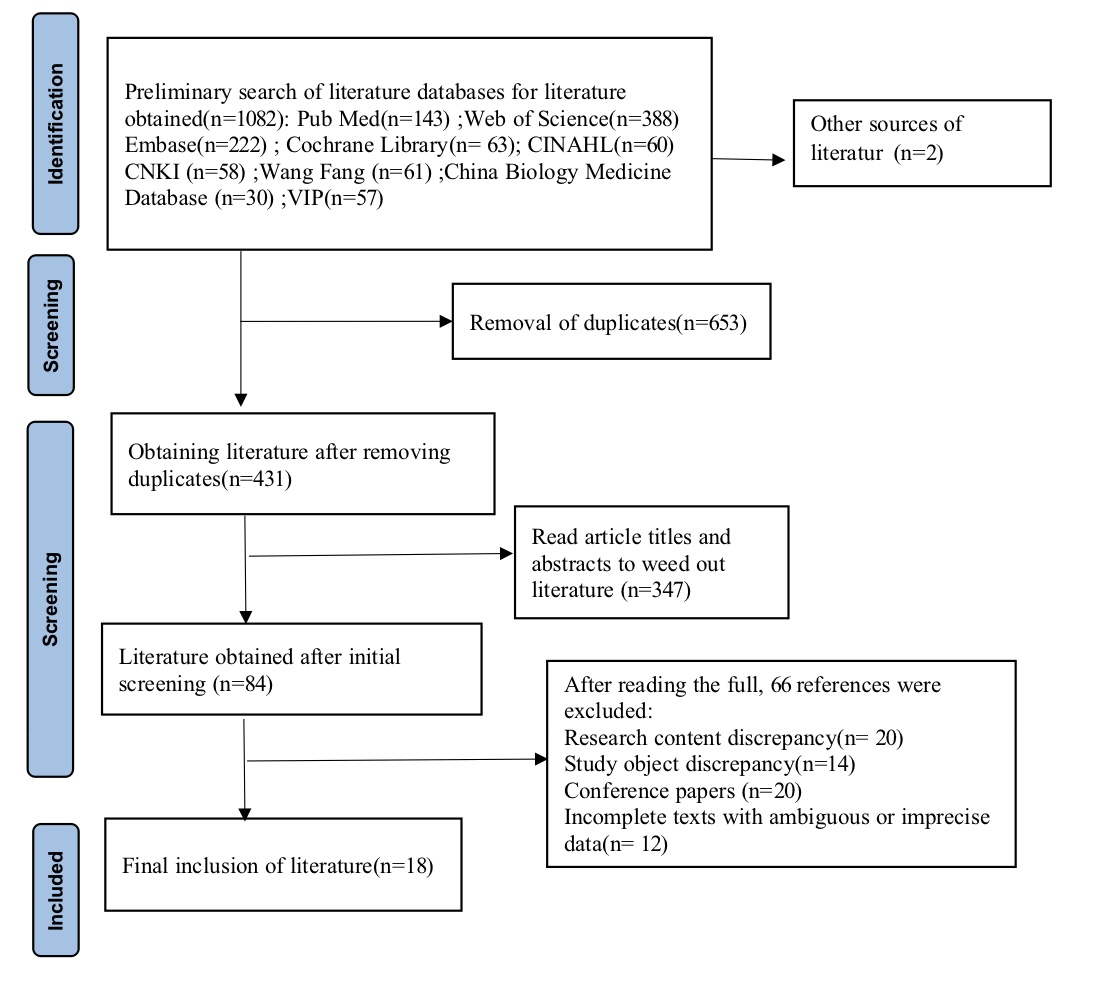

Supplement: S1 Fig — (JPG) [file pone.0313841.s003.jpg]
