# Supplementary material for: Application of clown care in hospitalized children: A scoping review
Source: PLoS One. 2024 Dec 19;19(12):e0313841. doi: 10.1371/journal.pone.0313841 (PMC11658477; doi:10.1371/journal.pone.0313841)
Supplement: S1 Table — (DOCX) [file pone.0313841.s004.docx]

**S1 Table****. General characteristics of the included studies（n=18）**

| **Reference** | **publication year** | **Country** | **Study Design** | **population** | **clowns**  **practitioner** | **Timing of intervention** | **Forms of intervention** | **conclusion**  **mark** |
| --- | --- | --- | --- | --- | --- | --- | --- | --- |
| Ben-Pazi H, et al.[16] | 2017 | Italy | Randomized controlled trial | A | Medical clowns | puncture therapy | Cognitive responses;  Empowerment;  Emotional reflection; | ① |
| Felluga M, et al.[17] | 2016 | Italy | quasi-randomized controlled trial | A | Medical clowns | puncture therapy | Cognitive responses；  Divert attention; | ①② |
| Goldberg A, et al.[18] | 2014 | Israel | Randomized controlled tria | A | Medical clowns | puncture therapy | Divert attention | ①② |
| Kristensen HN, et al.  [19] | 2018 | Denmark | Controlled clinical trial | A | Medical clowns | puncture therapy | Cognitive responses;  Divert attention; | ① |
| Meiri N, et al.[20] | 2015 | United States | Randomized controlled tria | A | Medical clowns | puncture therapy | Divert attention; | ①②④ |
| NingXuemei,et al.[21] | 2023 | China | Randomized controlled tria | A | Medical clowns | puncture therapy | Divert attention | ①②④ |

| Yang Fan, et al.[22] | | 2022 | China | Randomized controlled tria | A | Medical clowns | puncture therapy | Cognitive responses;  Divert attention;  Guided imagery; | ①④ |
| --- | --- | --- | --- | --- | --- | --- | --- | --- | --- |
| Dionigi A, et al.  [23] | 2014 | Italy | Randomized controlled tria | B | Medical clowns | perioperative period | Divert attention; | ① |  |
| Markova G,et al.  [24] | 2021 | Germany | Randomized controlled tria | B | Medical clowns | perioperative period | Guided imagery;  Divert attention; | ② |  |
| Vagnoli L, et al. [25] | 2005 | Italy | Randomized controlled tria | B | Medical clowns | perioperative period | Guided imagery；  Divert attention | ② |  |
| Cheng Zongyan, et al.  [26] | 2019 | China | Randomized controlled tria | B | Medical clowns | perioperative period | Cognitive responses;  Divert attention;  Guided imagery; | ①④ |  |
| Yildirim M, et al.  [27] | 2018 | Turkish | Randomized controlled tria | C | Medical clowns | dressing changes | Guided imagery;  Emotional reflection; | ③ |  |
| Yan Guifan, et al.  [28] | 2023 | China | Randomized controlled tria | C | Medical clowns | dressing changes | Divert attention; | ①④ |  |
| Wan Lin, et al  [29] | 2023 | China | Randomized controlled tria | D | Medical clowns | Postoperative fracture and rehabilitation | Divert attention;  Cognitive responses | ①④ |  |
| Lopes-Junior LC, et al  [30] | 2020 | Brazil | quasi-randomized controlled trial | E | volunteers | Before and after chemotherapy | Divert attention; | ②④ |  |
| Shefer S, et al.  [31] | 2019 | Israel | Controlled clinical trial | F | Medical clowns | When treating autism | Emotional reflection;  Cognitive responses | ③ |  |
| Bertini,et al.[32] | 2011 | Italy | Randomized controlled tria | G | Medical clowns | When the child was in the hospital room | Divert attention | ①④ |  |

| Shauna et al [33] | 2011 | Canada | non-randomised controlled trials | H | Medical clowns | When the child was in the hospital room | Divert attention  Empowerment; | ② |
| --- | --- | --- | --- | --- | --- | --- | --- | --- |

**Footnote**: A children with puncture therapy, B children in perioperative period, C children with burns, D children after fracture surgery, E children with chemotherapy, F children with autism;G Children hospitalized for respiratory pathologies, H disabled children,

①physiological indicators, ②psychological indicators, ③ cognitive-behavioral indicators, ④other indicators.
