# Supplementary material for: Application of clown care in hospitalized children: A scoping review
Source: PLoS One. 2024 Dec 19;19(12):e0313841. doi: 10.1371/journal.pone.0313841 (PMC11658477; doi:10.1371/journal.pone.0313841)
Supplement: Attachment — Submitted filename: PONE-D-24-35005_reviewer.docx [file pone.0313841.s005.docx]

**Title**

Application of clown care in hospitalized children: A scoping review

Xin Guo^1^, Yingping Fu^1,^ Yue Chen^1^, Jiaming Wu^1^, Xue Hu^2^*

^1^ School of nursing, Yunnan University of Chinese Medicine, No. 1076, Yuhua Road, Chenggong District, Kunming City, Yunnan Province,China,

^2^ Neonatology,Yan'an Hospital Affiliated to Kunming Medical University, No. 245, Renmin East Road, Kunming City, Yunnan Province, China.

* Corresponding author

E-mail: huxue123451@qq.com (XH)

**Abstract**

**Introduction**

Hospitalized children's developmental progress in emotional, behavioral, cognitive, and educational aspects could suffer due to their limited cognitive abilities and unfamiliarity with the hospital environment. Clown care has proven to be a successful non-pharmacological intervention in recent years for easing hospitalized children's concerns and anxieties and assisting them in adjusting to the hospital setting. Research on the efficacy of clown care for hospitalized children has produced varying results, with differences in treatment duration and methods.

This study aims to provide healthcare providers with insights into relevant interventions by examining the timing, format, content, outcome measures, and effects of clown care interventions on hospitalized children.

**Methods**

This study utilized a scoping review methodology based on the framework by Arksey and O’Malley. The search for Chinese and English literature on the utilization of clown care for hospitalized children was conducted in databases including CNKI, VIP, Wanfang, China Biology Medicine Database, PubMed, Embase, Web of Science, CINAHL, and Cochrane Library. At the same time, the references and other grey literatures included in the paper were also manually searched. covering publications from inception to December 10, 2023. Screening of the literature was done based on predefined inclusion and exclusion criteria, with data extraction and synthesis carried out independently by two researchers.

**Results**

A total of 16 articles were included. Clown care can be used with children who have burns, perioperative care, fracture rehabilitation, autism, puncture therapy, and chemotherapy, according to a review of the literature. The main interventions used in clown care are guided imagery, cognitive coping, emotional reflection, distraction, and empowerment. Physical, psychological, cognitive-behavioral, and other indications are the several types of clown care outcome indicators.

**Conclusions**

The study concludes that hospitalized children can benefit from clown care interventions, which exhibit diversity but lack specificity. Future research should concentrate on understanding the characteristics of hospitalized children to develop tailored clown care programs that can enhance clinical effectiveness.

**Keywords:**

**Trial registration number**

<https://doi.org/10.17605/OSF.IO/B4KMV>

**Introduction**

Hospitalization can have adverse effects on an individual's emotional, behavioral, cognitive, and educational development, particularly in children, due to their limited cognitive abilities and unfamiliarity with the hospital environment [1]. This can lead to feelings of fear and anxiety, hindering medical interventions and causing significant stress reactions [2]. Therefore, it is crucial to assist hospitalized children in acclimating to their new surroundings and implementing effective interventions to alleviate their concerns and anxieties. Clown care, also known as clown therapy or medical clowning, has emerged as a popular non-pharmacological intervention in pediatric care, geriatrics, and chronic disease management in recent years. Rooted in humor, clown care aims to help hospitalized patients manage stress, anxiety, and pain to expedite their recovery. Medical and nursing interventions incorporate elements of humor, juggling, magic, mime, storytelling, and music, with interventions tailored to each child based on their age, preferences, and clinical condition [3]. Studies have shown that offering clown care to hospitalized children can be a beneficial approach to reducing worry, fear, and discomfort associated with medical procedures, ultimately contributing to their recovery [4]. Clown care programs have been widely adopted internationally since the emergence of hospital clowns in North America in the 1980s; currently, clown doctor initiatives are available in various regions including the US, Canada, Europe, Israel, Australia, South Africa, Asia, and South America [5].

Clown care programs are still in the early stages of development in China, primarily due to cultural differences [6], and their implementation for hospitalized children is not yet widespread in the country [7]. Variations in staff qualifications, intervention protocols, and research methodologies exist, with limited relevant studies conducted in China. To provide healthcare professionals with guidance on relevant interventions, this study utilized a scoping review framework [8]. Literature from both domestic and international sources was reviewed and analyzed to outline the structure and content of clown care interventions, the optimal timing for interventions in hospitalized children, as well as the outcome indicators and effects.

**Methods**

This study follows the framework outlined by Arksey and O'Malley, which includes five essential stages: 1) Formulating the research question; 2) Identifying pertinent studies; 3) Selecting studies; 4) Organizing data, and; 5) Synthesizing, summarizing, and presenting findings.

**Stage one: Formulating the research question**

According to the principle of participants, concept, context (PCC), the study object is hospitalized children, the study concept is clown care, and the study context is the application of clown care in hospitalized children.

Finalizing research questions:

- What are the advantages and limitations of clown care in the application of hospitalized children?
- What are the factors influencing the application of clown care in hospitalized children?
- How can existing programs and application modalities aid hospitalized children in adapting to their new environment and alleviating negative emotions?
- The measures used by clown care in hospitalized children are as follows?

**Stage two: Identifying relevant studies**

A systematic search was conducted across databases such as CNKI, VIP, Wanfang, China Biology Medicine Database, PubMed, Embase, Web of Science, CINAHL, and Cochrane Library, aligning with the identified research queries. The search was restricted to articles published up to December 10, 2023. A comprehensive search strategy combining controlled vocabulary and free-text terms with Boolean operators "AND" and "OR" was employed. The search strategy tested in PubMed is presented in Table 1. Prior to screening, systematic training exercises were conducted on researchers to ensure the consistency of the studies. To address discrepancies, examiners revisit inclusion and exclusion criteria and perform additional screening. All related articles are managed using EndNote X9 Reference Manager, which also helps to identify duplicate entries.

Table 1 Pilot search in PubMed electronic database

| Date |  | Keywords | Search results | |
| --- | --- | --- | --- | --- |
| 10/12/2023 | #1Clown [Title/Abstract] OR medical clown [Title/Abstract] OR clown doctor [Title/Abstract] OR clown intervention [Title/Abstract] OR clown therapy [Title/Abstract] OR clown nurse [Title/Abstract]  #2child [Mesh Terms] OR children [Title/Abstract] OR pediatric [Title/Abstract] OR kids [Title/Abstract]  #3 #1AND#2 | | 1082 |  |

**Stage three: Study selection**

Eligibility and Inclusion Criteria

Inclusion criteria

- Hospitalized children, along with any accompanying family members
- Evaluation of the impact of clown care on hospitalized children, including medical clowns, clown doctors, clown nurses, etc.
- Original research literature, encompassing qualitative studies, case-control studies, cohort studies, case studies, etc.
- Language of publication in either Chinese or English.

Exclusion Criteria:

- Literature where the effect of clown care in combination with other interventions is challenging to quantify
- Conference papers or incomplete texts with ambiguous or imprecise data
- Duplicate publications
- Literature not available in Chinese or English.

Selection process

To eliminate duplicates, all literature was imported into the EndNote X9 software. Two researchers trained in evidence-based practice independently screened titles and abstracts based on the inclusion and exclusion criteria. Full-text review was conducted for all potentially relevant articles. In case of disagreements, a third researcher reviewed the material and a consensus was reached. The screening process is depicted in Fig 1.

Figure 1 Flow chart of literature screening

**Stage four: Charting the data**

A form was developed in Excel for the data extraction and piloted to ensure its accuracy. After carefully reading the entire text, the first and second researchers collected all pertinent data from the included literature. The following information was included in the data extraction form: the study's title, authors, nation, publishing date, kind of study, sample size, study population, intervention strategy, and outcome indicators. Once the data were plotted, a third researcher checked the data's quality and made any necessary updates to the extracted data to include all the data and improve the study's overall completion.

**Stage five: Collating, summarizing and reporting results**

The following was served as the foundation for themes pertaining to the study objectives: (i) What pediatric patients of clown care are provided to children in hospitals? (ii) In what ways and with what pediatric patients of interventions does clown care get applied to pediatric patients in hospitals? (iii) How does providing clown care to pediatric patients in hospitals affect their evaluation? This will function as a story with pertinent themes. To the extent feasible, figures and tables will also be utilized to present the findings.

**Results**

**Characteristics of the studies selected**

After removing 653 duplicate entries from the initial 1082 pieces of literature identified through the stepwise search, 429 pieces remained. Following a review of titles and abstracts, 342 articles were excluded. Subsequently, 71 articles that did not meet the inclusion criteria were eliminated after full-text assessment, leaving 16 articles for final inclusion. Among these, 11 were in English and 5 were in Chinese. The included studies originated from countries such as Italy [9,10,16,18], Israel [11,24], Denmark [12], Brazil [23], Germany [17], the United States [13], Turkey [20], and China [14,15,19,21,22] are among the countries whose works were published between 2005 and 2023 (Table 2).

Table 2 General characteristics of the included studies（n=16）

| **Reference** | **publication year** | **Country** | **Study Design** | **population** | **clowns**  **practitioner** | **Timing of intervention** | **Forms of intervention** | **conclusion**  **mark** |
| --- | --- | --- | --- | --- | --- | --- | --- | --- |
| Ben-Pazi H, et al.[9] | 2017 | Italy | Randomized controlled trial | A | Medical clowns | puncture therapy | Cognitive responses;  Empowerment;  Emotional reflection; | ① |
| Felluga M, et al.[10] | 2016 | Italy | quasi-randomized controlled trial | A | Medical clowns | puncture therapy | Cognitive responses；  Divert attention; | ①② |
| Goldberg A, et al.[11] | 2014 | Palestine | Randomized controlled tria | A | Medical clowns | puncture therapy | Divert attention | ①② |
| Kristensen HN, et al.  [12] | 2018 | Denmark | Controlled clinical trial | A | Medical clowns | puncture therapy | Cognitive responses;  Divert attention; | ① |
| Meiri N, et al.[13] | 2015 | United States | Randomized controlled tria | A | Medical clowns | puncture therapy | Divert attention; | ①②④ |
| NingXuemei,et al.[14] | 2023 | China | Randomized controlled tria | A | Medical clowns | puncture therapy | Divert attention | ①②④ |
| Yang Fan, et al.[15] | 2022 | China | Randomized controlled tria | A | Medical clowns | puncture therapy | Cognitive responses;  Divert attention;  Guided imagery; | ①④ |
| Dionigi A, et al.[16] | 2014 | Italy | Randomized controlled tria | B | Medical clowns | perioperative period | Divert attention; | ① |
| Markova G,et al.[17] | 2021 | Germany | Randomized controlled tria | B | Medical clowns | perioperative period | Guided imagery;  Divert attention; | ② |

Continued Table 2 General characteristics of the included studies（n=16）

| **Reference** | **publication year** | **Country** | **Study Design** | **population** | **clowns**  **practitioner** | **Timing of intervention** | **Forms of intervention** | **conclusion**  **mark** |
| --- | --- | --- | --- | --- | --- | --- | --- | --- |
| Vagnoli L, et al. [18] | 2005 | Italy | Randomized controlled tria | B | Medical clowns | perioperative period | Guided imagery；  Divert attention | ② |
| Cheng Zongyan, et al.[19] | 2019 | China | Randomized controlled tria | B | Medical clowns | perioperative period | Cognitive responses;  Divert attention;  Guided imagery; | ①④ |
| Yildirim M, et al.  [20] | 2018 | Turkish | Randomized controlled tria | C | Medical clowns | dressing changes | Guided imagery;  Emotional reflection; | ③ |
| Yan Guifan, et al.  [21] | 2023 | China | Randomized controlled tria | C | Medical clowns | dressing changes | Divert attention; | ①④ |
| Wan Lin, et al.[22] | 2023 | China | Randomized controlled tria | D | Medical clowns | Postoperative fracture and rehabilitation | Divert attention;  Cognitive responses | ①④ |
| Lopes-Junior LC, et al.[23] | 2020 | Brazil | quasi-randomized controlled trial | E | volunteers | Before and after chemotherapy | Divert attention; | ②④ |
| Shefer S, et al.[24] | 2019 | Palestine | Controlled clinical trial | F | Medical clowns | When treating autism | Emotional reflection;  Cognitive responses | ③ |

**Footnote**: A children with puncture therapy, B children in perioperative period, C children with burns, D children after fracture surgery, E children with chemotherapy, F children with autism; ①physiological indicators, ②psychological indicators, ③ cognitive-behavioral indicators, ④other indicators.

**Essential Elements of Clown Care**

**Clown Care Clients and Clown Care Practitioners**

Clown care is utilized in various settings for hospitalized children, including acupuncture therapy [9,10,11,12,13,14,15], perioperative [16,17,18,19], burn treatment [20,21], fracture rehabilitation [22], chemotherapy support [23], and autism intervention [24]. Evaluation of clown care often involves measuring parent satisfaction, caregiver anxiety levels, and caregiver trait anxiety in the context of hospitalized children [11, 14,15, 16,18, 21, 22].

Individuals responsible for designing and implementing clown care programs are referred to as clown care practitioners in this study. These practitioners primarily consist of medical clowns who have received training in sociology, psychology, pedagogy, acting, nursing, and healthcare [10,11,12,13,14,15,16,17,18,19,20,21,22,23,24]. Additionally, clown care volunteers, including clown doctors, clown nurses, and professional medical clowns, are also included within this category.

**Timing of clown care intervention for hospitalized children**

Among the sixteen studies compiled, seven studies [9,10,11,12,13,14,15] discussed the application of clown care in puncture therapy, encompassing procedures like venepuncture, intravenous infusions, phlebotomy, botulinum toxin injections, and allergy prick testing conducted during the hospital stay. Four publications [16,17,18,19,20] focused on the use of clown care in anesthesia for induction, postoperative care, and preoperative care in the perioperative period. Two studies [20,21] examined discomfort related to burns on the body, emphasizing pre-procedure anxiety, discomfort during dressing changes, and post-procedural relaxation. Postoperative rehabilitation following a fracture was explored in one paper [22], covering both postoperative fracture management and rehabilitation. Another paper [23] discussed the application of clown care during chemotherapy sessions. Additionally, one study [24] investigated the provision of clown care to children with autism, focusing on the timing of interventions ten minutes before, during, and after the procedure.

**Form and content of clown care**

The elements of clown care can be encapsulated by terms such as "cognitive coping," "guided imagery," "distraction," "emotional reflection," and "empowerment." "Cognitive coping" [9,10,12,15,19,22,24] involves establishing a rapport with the child before the procedure, encouraging the child to manage challenges posed by illness constructively, promoting self-awareness[25], and acknowledging the child's courage in facing difficulties. "Guided imagery" [9,15,17,18,19,20,23] entails using clowning to create positive memories and help children cope with pain by guiding them to imagine pleasant experiences. "Distraction" [10,11,12,13,14,15,16,17,18,19,20,21,22,23] involves medical clowns dressing up as beloved characters to capture the children's attention and employing magic, mime, storytelling, music, and other diversions tailored to the children's preferences to reduce focus on discomfort and anxiety during procedures. "Emotional reflection" [9,19,20,24] involves adapting the clown's performance to the child's emotional cues, engaging with the child in a manner that resonates with their feelings, and encouraging the child to express themselves during the interaction. "Empowerment" [9] involves the clown adjusting their actions based on the child's input, fostering the child's confidence and agency. By simulating medical procedures as a game, children gain a better understanding of the process, reducing anxiety and fear.

**Outcome indicators and effects**

The collection of sixteen studies outlined five sets of outcome measures: behavioral, cognitive-behavioral, physical, and miscellaneous markers. Physiological indicators were reported in ten studies [9,10,11,12,13,14,15,19,21,22] focusing primarily on children's pain levels (measured using various scales such as Pain Visual Analog Scale, Pain Ruler, Numeric Pain Rating Scale, Facial Expression Pain Inventory, FLACC Pain Assessment Scale, self-reported pain levels, modified Facial Pain Expression Scale known as Piggyback), children's fatigue (assessed using Pediatric Quality of Life Scale, Child Multidimensional Fatigue Scale), duration of children's crying, healing times for fractures (using Femadez-esteve scale), and improvement in joint function (measured with Flynn scale). Psychological markers were reported in seven studies [10,11,14,16,17,18,23] focusing on children's positive emotions, caregiver anxiety levels (State-Trait Anxiety Inventory), children's emotional states and anxiety levels (Modified Yale Preoperative Anxiety Scale), trait anxiety (Children's State-Trait Anxiety Inventory), and psychological stress (Children's Stress Inventory). Cognitive-behavioral indicators were discussed in three studies [20,21,24], highlighting metrics such as the number of meaningful words spoken in ten minutes, instances of social smiles (Autism Diagnostic Scale, Second Edition), interactions during a game of "catch," and compliance with medication regimens. Additional measures, including salivary cortisol levels, duration of child's crying, amount of pain medication used within 24 hours post-surgery, success rate of medical procedures, and parental satisfaction (assessed through questionnaires), were detailed in eight studies [13,14,15,19,20,21,22,,21,22,23].

**Discussion**

**Clown care interventions are varied, but programs lack individualization**

The study findings suggest that while there exists a wide range of clown care interventions, the predominant approaches revolve around "cognitive coping," "guided imagery," "distraction," "emotional reflection," and "empowerment." Clown phobia refers to the extreme aversion and distress experienced by individuals towards clown figures. Each patient is a unique individual with distinct characteristics. In a study involving 1160 children receiving clown care interventions, 14 of them exhibited fear or phobia towards medical clowns [26]. The onset of these phobic reactions in children was found to be unrelated to factors such as race, religion, specific illnesses, or associated symptoms. Research indicates that symptoms of clown phobia can persist for up to 60 years [27]. Furthermore, 89.5% of patients reported no significant improvement in their fear of clowns, nor did it alleviate social or psychiatric issues. Children may lack the cognitive capacity, and their hospitalization can already induce significant stress. Incorrectly tailored clown care interventions based on the children's individual traits could lead to lasting psychological harm and hinder the normal development of their psychological, physiological, and cognitive functions. To enhance the efficacy of clown care programs and tailor them more effectively, it is crucial to integrate the unique needs and characteristics of hospitalized children into these interventions. Clinical practitioners should assess high-risk children for clown phobia before implementing clown care to prevent potential harm and address underlying issues. Further research is needed to enhance the focus, effectiveness, and clinical relevance of clown care for hospitalized children. Currently, there is a lack of assessment tools specifically designed for pediatric clown care.

**A lesser known study in clown care examines the effect on biochemical tests such as salivary cortisol concentration in hospitalized children**

To effectively manage and alleviate the pain experienced by hospitalized children, it is essential to conduct a thorough and precise evaluation of their discomfort. As some hospitalized children may struggle to articulate their pain levels, healthcare professionals often rely on observational scales to assess pain based on the children's behaviors, such as limb movements and facial expressions [28]. However, it has been suggested [29] that pain assessment tools focusing on behavioral and facial cues might be limited to subcortical somatic and autonomic motor pathways, which may not accurately capture a child's pain experience. Cortisol, the primary stress hormone produced by the body, has emerged as a valuable objective marker for evaluating children's discomfort and the effectiveness of treatments [30]. Saliva samples are easy to collect and non-invasive, and salivary cortisol represents biologically active free cortisol [31], enhancing the precision of assessments. Cortisol levels can be measured through blood, saliva, and urine samples. Among the 16 studies included in this investigation, only one [23] utilized salivary cortisol as an outcome measure to assess pain in children. To provide scientific validation for the advancement of clown care programs that effectively alleviate pain, reduce physical and psychological distress, shorten hospital stays, and aid in recovery, integrating pain assessment scales with biochemical testing in future research and clinical applications can offer valuable insights.

**Exploring cost-effective and convenient alternatives to on-site clown care provision**

The implementation of clown care clinical activities for hospitalized children was carried out on-site in the 16 publications included in this study. The healthcare landscape is evolving, with increasing demands on healthcare professionals as societal advancements and healthcare models undergo updating and transformation. Pediatric wards entail high-risk, high-intensity, and high-pressure medical work compared to other departments. Introducing clown care may add to the workload of medical professionals. Moreover, the intense stress of clinical work could potentially lead to professional burnout and impact clinical outcomes for children [32]. Conversely, establishing a clown care team requires a substantial number of healthcare experts; therefore, virtual reality can be leveraged to enhance clinical tasks related to clown care interventions [33]. This approach can aid in the efficient allocation of medical resources, potentially leading to a more equitable distribution of human resources among clinical healthcare specialists. In addition to alleviating the burden on medical staff and enhancing treatment adherence for hospitalized children, virtual reality (VR) technology presents itself as a cutting-edge, cost-effective, and practical means to deliver care to children in hospital settings. By integrating virtual reality technology with clown care, such as offering a virtual reality interactive platform featuring children's favorite cartoons to aid in engagement, we can provide a more immersive and tailored care experience. The utilization of virtual reality technology in healthcare is expanding and has shown promise in various domains, including pain management [34], psychotherapy [35], and other areas. It is recommended that future research explores more affordable and convenient programs that leverage virtual reality technology to enhance care delivery.

**Clown care should be culturally appropriate and localized**

The traditional Western clown appearance, characterized by white faces, red noses, and black eyes, may not be suitable for hospitalized children in China and many other Eastern countries due to cultural differences, potentially causing fear instead of comfort. Chinese children in hospitals may not respond positively to clown care interventions that involve mime, puppetry, or similar performance-based techniques. While some studies have demonstrated the benefits of clown care in alleviating pain and anxiety in children from diverse cultural backgrounds [35], it is evident that children from varying cultural backgrounds may respond differently to the same clown care approaches, underscoring the influence of cultural values and backgrounds on the effectiveness of such interventions. Therefore, it is essential to carefully consider and incorporate the cultural values and norms of each nation when implementing clown care for hospitalized children from different cultural backgrounds. Developing a culturally localized clown care strategy specific to our country is imperative.

**Limitations of the Study**

Acknowledging the constraints of a scoping review is crucial. To begin with, the process of selecting articles did not involve any quality criteria. While this is not a mandatory methodological requirement for conducting a scoping review, it does hinder the authors' ability to evaluate the reliability and strength of the literature included, consequently impacting the confidence in the conclusions drawn from it. Additionally, the generalizability of the findings is restricted as we solely incorporated published works in both Chinese and English during the selection of literature for inclusion.

**Conclusion**

Children in hospital settings experience significant benefits from clown care interventions that positively impact their physical, mental, and cognitive well-being. This study delved into the scope of clown care initiatives for pediatric patients in hospitals, systematically categorizing research subjects, practitioners, intervention modalities, content, and outcome measures associated with clown care. The results highlighted the favorable and successful effects of implementing clown care for children in hospital settings. Despite the existence of diverse clown care interventions, they lacked personalization. Additionally, the utilization of physiological markers such as salivary cortisol was infrequent, with measures and questionnaires being the predominant assessment tools for evaluating the impact of clown care. To establish a robust and scientifically grounded framework for the clinical advancement of high-quality clown care programs for hospitalized children, future research should explore the influence of various cultural backgrounds and values.

**Supporting information**

**S1 File. PRISMA-ScR checklist.**

(DOCX)

**Author Contributions**

**Conceptualization:** Xin Guo, Yingping Fu.

**Data curation:** Xin Guo, Yue Chen, Jiaming Wu.

**Formal analysis:** Xin Guo, Yingping Fu, Xue Hu, Yue Chen, Jiaming Wu.

**Methodology:** Xin Guo, Yingping Fu.

**Project administration:** Xin Guo, Yingping Fu, Xue Hu.

**Supervision:** Xin Guo, Yingping Fu, Xue Hu.

**Writing – original draft:** Xin Guo, Yingping Fu, Xue Hu, Yue Chen, Jiaming Wu.

**Writing – review & editing:**	Xin Guo, Yingping Fu, Xue Hu, Yue Chen, Jiaming Wu.

**Funding Statement**

The author(s) received no specific funding for this work.

**References**

[1]Barkmann C, Siem AK, Wessolowski N, Schulte-Markwort M. Clowning as a supportive measure in paediatrics - a survey of clowns, parents and nursing staff. BMC Pediatr. 2013 Oct 10;13:166. doi: 10.1186/1471-2431-13-166. PMID: 24112744; PMCID: PMC3851858.
[2]Bray L, Appleton V, Sharpe A. The information needs of children having clinical procedures in hospital: Will it hurt? Will I feel scared? What can I do to stay calm? Child Care Health Dev. 2019 Sep;45(5):737-743. doi: 10.1111/cch.12692. Epub 2019 Jul 18. PMID: 31163093; PMCID: PMC6851850.
[3]Finlay F, Baverstock A, Lenton S. Therapeutic clowning in hospital settings: more than a play-fool proposal. Arch Dis Child. 2017 Jul;102(7):597-598. doi: 10.1136/archdischild-2017-312715. Epub 2017 May 2. PMID: 28465302.
[4]Yildirim M, Koroglu E, Yucel C, Kirlak S, Sen S. The effect of hospital clown nurse on children's compliance to burn dressing change. Burns. 2019 Feb;45(1):190-198. doi: 10.1016/j.burns.2018.08.033. Epub 2018 Sep 21. PMID: 30249435.
[5]Lopes-Júnior LC, Bomfim E, Olson K, et al. Effectiveness of hospital clowns for symptom management in paediatrics: systematic review of randomized and non-randomized controlled trials. BMJ. 2020 Dec 16;371:m4290. doi: 10.1136/bmj.m4290. PMID: 33328164; PMCID: PMC7737653.
[6]YU Xiaoyan, LIU Yu, HUANG Yi. Effects of Clown Care on Children's Anxiety and Pain: A Meta-analysis. Journal of Nursing（China）.2022, 29 (04): 21-26. DOI:10.16460/j.issn1008-9969.2022.04.021.
[7]Wang Pei, Zhang Lanfang, Cui Yi, Zhang Yinling. Developments in the field of research on clown care for young patients. Chinese Journal of Nursing.2023, 58 (08): 1012-1016.
[8]Peters MD, Godfrey CM, Khalil H, McInerney P, Parker D, Soares CB. Guidance for conducting systematic scoping reviews. Int J Evid Based Healthc. 2015 Sep;13(3):141-6. doi: 10.1097/XEB.0000000000000050. PMID: 26134548.
[9]Ben-Pazi H, Cohen A, Kroyzer N, Lotem-Ophir R, Shvili Y, Winter G, Deutsch L, Pollak Y. Clown-care reduces pain in children with cerebral palsy undergoing recurrent botulinum toxin injections-A quasi-randomized controlled crossover study.PLoS One. 2017 Apr 17; 12(4):e0175028. doi: 10.1371/journal.pone.0175028. PMID: 28414728; PMCID: PMC5393564.
[10]Felluga M, Rabach I, Minute M, Montico M, Giorgi R, Lonciari I, Taddio A, et al. A quasi randomized-controlled trial to evaluate the effectiveness of clowntherapy on children's anxiety and pain levels in emergency department. Eur J Pediatr. 2016 May;175(5):645-50. doi: 10.1007/s00431-015-2688-0. Epub 2016 Jan 12. PMID: 26755209.
[11]Goldberg A, Stauber T, Peleg O, Hanuka P, Eshayek L, Confino-Cohen R. Medical clowns ease anxiety and pain perceived by children undergoing allergy prick skin tests. Allergy. 2014 Oct; 69(10):1372-9. doi: 10.1111/all.12463. Epub 2014 Aug 4. PMID: 24943088.
[12]Kristensen HN, Lundbye-Christensen S, Haslund-Thomsen H, Graven-Nielsen T, Elgaard Sørensen E. Acute Procedural Pain in Children: Intervention With the Hospital Clown. Clin J Pain. 2018 Nov;34(11):1032-1038. doi: 10.1097/AJP.0000000000000625. PMID: 29727302.
[13]Meiri N, Ankri A, Hamad-Saied M, Konopnicki M, Pillar G. The effect of medical clowning on reducing pain, crying, and anxiety in children aged 2-10 years old undergoing venous blood drawing--a randomized controlled study. Eur J Pediatr. 2016 Mar; 175(3):373-9. doi: 10.1007/s00431-015-2652-z. Epub 2015 Oct 16. PMID: 26475347.
[14]Ning Xuemei, Jiang Wenxiu, Qiu Ting, Liu Zhanli, Li Ruilan, Sha Sha. Effect of Therapeutic Clowning on Venous Blood Sampling and Parental Satisfaction of Hospitalized Preschoolers. Journal of Chengdu Medical College. 2023, 18 (02): 253-256.
[15]Yang Fang, Zheng Silin, Jiang Li, Yi Zihan, Liu Jiru, Zhang Yan, Tao Li,Peng Hong. Application of clown care in intravenous infusion puncture among preschool children. Journal of Nursing（China）. 2022, 37 (06): 12-14.
[16]Dionigi A, Sangiorgi D, Flangini R. Clown intervention to reduce preoperative anxiety in children and parents: a randomized controlled trial. J Health Psychol. 2014 Mar;19(3):369-80. doi: 10.1177/1359105312471567. Epub 2013 Jan 29. PMID: 23362335.
[17]Markova G, Houdek L, Kocabova Z. To the Operating Room! Positive Effects of a Healthcare Clown Intervention on Children Undergoing Surgery. Front Public Health. 2021 Apr 20;9:653884. doi: 10.3389/fpubh.2021.653884. PMID: 33959583; PMCID: PMC8093515.
[18].Vagnoli L, Caprilli S, Robiglio A, Messeri A. Clown doctors as a treatment for preoperative anxiety in children: a randomized, prospective study. Pediatrics. 2005 Oct;116(4):e563-7. doi: 10.1542/peds.2005-0466. PMID: 16199685.
[19]CHENG Zongyan, CAO Qing, ZHANG Ling, LIAO Fengqin, HE Lin. Clinical clownish intervention for crying and pain in children with perianaesthesia. Medical Journal of West China. 2019, 31 (04): 629-632.
[20]Yildirim M, Koroglu E, Yucel C, Kirlak S, Sen S. The effect of hospital clown nurse on children's compliance to burn dressing change. Burns. 2019 Feb;45(1):190-198. doi: 10.1016/j.burns.2018.08.033. Epub 2018 Sep 21. PMID: 30249435.
[21]Yan Guifang, Liang Qiong，Liu Yueming，Zhang Kun. Application of Modified Medical Clown Service in Burned Children Receiving Dressing Changes. Journal of Chengdu Medical College. 2023, 18 (05): 595-598.
[22]WANG Ling，YI Yu， HUANG Yuting，CHEN Bo，WANG Yuanhui，ZHANG Daiying. Clown care on postoperative rehabilitation of children with supracondylar fracture of humerus. Journal of Nursing Administration. 2023, 23 (01): 71-75.
[23]Lopes-Junior LC, Silveira DSC, Olson K, Bomfim EO, Veronez LC, Santos JC, Alonso JB, Nascimento LC, Pereira-da-Silva G, et al. Clown Intervention on Psychological Stress and Fatigue in Pediatric Patients With Cancer Undergoing Chemotherapy. Cancer Nurs. 2020 Jul/Aug;43(4):290-299. doi: 10.1097/NCC.0000000000000690. PMID: 30801267.
[24]Shefer S, Leon Attia O, Rosenan R, Wald OA, Ende H, Gabis LV. Benefits of medical clowning in the treatment of young children with autism spectrum disorder. Eur J Pediatr. 2019 Aug;178(8):1283-1289. doi: 10.1007/s00431-019-03415-7. Epub 2019 Jun 26. PMID: 31243575.
[25]LIAO Jianling，HE Jieping. Observation on the Application Effect of “Clown” Mode Nursing Method in Pediatric Ward. Medical Innovation of China. 2018, 15 (16）：087-090.
[26]Meiri N, Schnapp Z, Ankri A, Nahmias I, Raviv A, Sagi O ,et al. Fear of clowns in hospitalized children: prospective experience. Eur J Pediatr. 2017 Feb;176(2):269-272. doi: 10.1007/s00431-016-2826-3. Epub 2016 Dec 14. PMID: 27966019.
[27]Planting T, Koopowitz SM, Stein DJ. Coulrophobia: An investigation of clinical features. S Afr J Psychiatr. 2022 Jan 19;28:1653. doi: 10.4102/sajpsychiatry.v28i0.1653. PMID: 35169508; PMCID: PMC8831965.
[28]CHENG Rui, YANG Yang, SHI Yuan. Expert consensus on neonatal pain assessment and analgesia management (2020 edition). Chin J Contemp Pediat. 2020, 22 (09): 923-930.
[29]Slater R, Cantarella A, Franck L, Meek J, Fitzgerald M. How well do clinical pain assessment tools reflect pain in infants? PLoS Med. 2008 Jun 24;5(6):e129. doi: 10.1371/journal.pmed.0050129. PMID: 18578562; PMCID: PMC2504041.
[30]Jansen J, Beijers R, Riksen-Walraven M, de Weerth C. Cortisol reactivity in young infants. Psychoneuroendocrinology. 2010 Apr;35(3):329-38. doi: 10.1016/j.psyneuen.2009.07.008. Epub 2009 Aug 3. PMID: 19651476.
[31]Blair J, Adaway J, Keevil B, Ross R. Salivary cortisol and cortisone in the clinical setting. Curr Opin Endocrinol Diabetes Obes. 2017 Jun;24(3):161-168. doi: 10.1097/MED.0000000000000328. PMID: 28375882.
[32]Ding Qingmei, Bi Lexuan, Liu Na, Fang Xiaoqing, Liu Yaxin, Ding Xuemei, Zhang Xiaoli. Analysis of the current status and influencing factors of burnout among nurses in pediatric wards. Chinese Journal of Hospital Statistics. 2023,30(05):345-352+358.
[33]KONG Yun, ZHONG Kelong, AN Xuemei. Effect of virtual reality technology on improving motor function in stroke patients : a Meta-analysis. Chinese Evidence-Based Nursing. 2023, 9 (22): 3991-3997.
[34]Hoffman HG, Doctor JN, Patterson DR, Carrougher GJ, Furness TA 3rd. Virtual reality as an adjunctive pain control during burn wound care in adolescent patients. Pain. 2000 Mar;85(1-2):305-9. doi: 10.1016/s0304-3959(99)00275-4. PMID: 10692634.
[35]Wiebe A, Kannen K, Selaskowski B, Mehren A, Thöne AK, Pramme L, et al. Virtual reality in the diagnostic and therapy for mental disorders: A systematic review. Clin Psychol Rev. 2022 Dec;98:102213. doi: 10.1016/j.cpr.2022.102213. Epub 2022 Oct 30. PMID: 36356351.
