# Supplementary material for: Application of clown care in hospitalized children: A scoping review
Source: PLoS One. 2024 Dec 19;19(12):e0313841. doi: 10.1371/journal.pone.0313841 (PMC11658477; doi:10.1371/journal.pone.0313841)
Supplement: Attachment — Submitted filename: Response to Reviewers.docx [file pone.0313841.s006.docx]

**Response to Reviewers**

Dear Editor and Reviewers:

Greetings!

First of all, on behalf of all the authors, please allow me to extend our sincerest thanks to you and the reviewers. We would like to thank you for taking the time out of your busy schedules to review our paper in detail and provide valuable comments and suggestions. We are fully aware of the importance of these comments in improving the quality of the paper and ensuring academic rigor.

After receiving the review comments, we immediately organized our team to conduct in-depth discussion and analysis, and revised and improved the paper in strict accordance with the reviewers' suggestions. Editorial comments are listed below in red font. Reviewer comments are in blue font, such as below, where specific questions are numbered. Our responses are given in black font. The following are our responses and explanations to the reviewers' comments:

1. Please ensure that your manuscript meets PLOS ONE's style requirements, including those for ﬁle naming:

In order to comply with the strict formatting requirements of PLOS ONE, we have revised the formatting of the previous manuscript, which now conforms to PLOS ONE style, as described under "Revised Manuscript with Track Changes" and "Manuscript."

2. Please include captions for your Supporting Information files at the end of your manuscript, and update any in-text citations to match accordingly:

We have included the title of this research supporting information file at the end of the manuscript in strict accordance with the editor's request, and updated any textual citations accordingly,as described under "Revised Manuscript with Track Changes" and " Manuscript."

3. We note that your Data Availability Statement is currently as follows:”All relevant data are within the manuscript and its Supporting Information files.”Please confirm at this time whether or not your submission contains all raw data required to replicate the results of your study. Authors must share the “minimal data set” for their submission. PLOS ONE defines the minimal data set to consist of the data required to replicate all study findings reported in the article, as well as related metadata and methods:

In accordance with the requirements of PLOS ONE's Data Availability Statement and the recommendations of the editors, we have examined all of the raw data used in this study and assure you that our paper contains all of the raw data necessary to replicate the results of the study, as described in Table 1、Table 2 and Figure 1 of the manuscript and in the Supporting Information (S1 File、S2 File、S3 Fig、S4 Table.).

4. INTRODUCTION：There are some reviews about medical clowning for sick children, how does this article differ from them：

Thank you very much for your valuable comment. Based on your advice, we searched two other similar articles on clown care (https://doi.org/10.1007%2Fs12519-023-00720-y and https://doi.org/10.3389%2Ffped.2024.1324283). By carefully reading the full text, we have detailed the differences between the two articles and this study, as follows.

The purpose of article 1 (https://doi.org/10.1007%2Fs12519-023-00720-y) is to analyze the effects of clown care on hospitalized children. The findings suggest that clown care can reduce children's distress, relieve their anxiety, shorten their crying time, and raise caregivers' anxiety levels. However, some young children may be frightened by clowns and become tearful or anxious. The clown should be trained to notice and understand these children, and staff and parents should be consulted to help reduce anxiety until the particular child is satisfied with the clown. This is consistent with the results of this study. The purpose of article 2 (https://doi.org/10.3389%2Ffped.2024.1324283) was to evaluate the effectiveness of medical clowns in reducing pain and anxiety in hospitalized pediatric patients and their parents in different areas of medicine. The findings suggest that medical clowns have substantial positive and beneficial effects on reducing stress and anxiety in pediatric children and their families in a variety of settings, consistent with the findings of this study.

However, different from these two articles, the purpose of this study is to summarize the timing, form, and content of intervention, outcome indicators, and effects of clown care in hospitalized children. The first two articles only analyzed the application effect of clown care on hospitalized children but did not analyze the intervention timing, intervention form and content, and outcome indicators of clown care in different diseases. At the same time, the pain assessment in the above two articles may be limited to the pain assessment scale based on facial expression and behavior due to the incomplete literature included, while this study noted through literature analysis that the pain assessment tools based on facial expression and behavior may be limited to the measurement of subcortical body and autonomic motor pathways. This does not objectively diagnose the pain experience of the child. Cortisol is the main hormone in the body under stress, which can effectively detect pain in children , the research objects included in the first two articles are mostly children receiving invasive medical operations and surgeries, and this study also includes children with autism and children with cancer, so the research objects are more abundant, and the scope of clown care is wider.

In short, other articles on clown care mostly focused on analyzing the clinical application effect of clown care in hospitalized children. However, compared with other similar articles on clown care, this study not only analyzed the application effect of clown care but also fully analyzed and summarized its intervention timing, intervention form and content, and outcome indicators. In order to provide a reference for medical personnel to carry out relevant intervention.See "Manuscript with Track Changes" and "Manuscript".

5. INTRODUCTION：Why conduct a scoping review rather than other types of reviews：

We appreciate the editor's advice, which has been very helpful to us, and the following is what we added:

Many reviews have clarified the effectiveness of the application of clown care in pediatric patients, but there is a lack of comprehensive descriptions of the intervention timing, form and content of intervention, and outcome indicators of existing clown care. A scope review can summarize a range of documentary evidence to show the breadth and depth of a knowledge domain. Therefore, guided by the scope review report framework, this study summarized and analyzed the relevant literature in this field at home and abroad, and summarized the intervention timing, form and content of intervention, outcome indicators, and effects of clown care in hospitalized children, with a view to providing references for medical staff to carry out relevant research.

6. METHODS：For Eligibility criteria: Please state in PICOS：

Thank you very much for your valuable comment. Based on your advice, we changed the Eligibility criteria to PICOS format. Population (P): Hospitalized children < 18 years of age, along with any accompanying family members; Intervention (I): Receiving hospital clowns intervention; Comparison (C): Compared receipt of hospital clowns to standard of care; Outcome (O): The application and effect evaluation of clown care in hospitalized children; Study design (S): Original research literature, encompassing qualitative studies, case-control studies, cohort studies, case studies, etc. See the manuscript for details.

7. A similar meta-analysis review was conducted and completed in November 2019 and they yielded 14 studies. However, your review is a scoping review and should include more papers. Please revisit if you missed any potential research:

We strongly agree with the editor's view.In order to guarantee the scientific soundness and comprehensiveness of the scope review outcomes,we have strictly followed the editor's comments,and researched relevant studies on clown care and screened and read articles according to the inclusion and exclusion criteria in this paper. We have meticulously reviewed and incorporated several articles that are closely aligned with our research objectives and methodologies. We believe this will enhance the discourse and contextual framework of our study. Interestingly, we found two articles from other types of reviews, the last two papers in Table 2 ("Bertini et al[25], 2011"; "Shauna et al[26], 2011"). The research objects of these two articles have not been included in this study (the research objects are children hospitalized for respiratory pathologies and disabled children, respectively), and new information has been extracted from these two articles. For specifics, refer to the manuscript.We trust that the updated manuscript will fulfill your expectations.

8. METHODS：The discussion addresses the key points and limitations of the current study. However, it could be strengthened by 1) a more explicit comparison of the findings with the existing literature. 2) elaborate the implications for practice and policy; 3) provide more specific recommendations for future research:

We have meticulously updated the discussion section in strict alignment with the editor's recommendations and provided additional clarifications corresponding to each suggestion made by the editor. as described under "Revised Manuscript with Track Changes" and " Manuscript."

**Reviewer 1:**

Regarding Reviewer 1's comments, we are very grateful to the reviewer for taking the time to read our paper. We would like to thank you for your professional review work, constructive comments, and valuable suggestions on our manuscript. Your time and efforts are greatly appreciated. We have revised the manuscript according to your comments as listed in detail below.

1.Clearly state your research difference from other similar studies in 2023 and 2024(https://doi.org/10.1007%2Fs12519-023-00720-y, and https://doi.org/10.3389%2Ffped.2024.1324283):

Thank you very much for your valuable comment. Based on your advice,we have read both articles on clown care (https://doi.org/10.1007%2Fs12519-023-00720-y and https://doi.org/10.3389%2Ffped.2024.1324283). By carefully reading the full text, we have detailed the differences between the two articles and this study, as follows.

The purpose of article 1 (https://doi.org/10.1007%2Fs12519-023-00720-y) is to analyze the effects of clown care on hospitalized children. The findings suggest that clown care can reduce children's distress, relieve their anxiety, shorten their crying time, and raise caregivers' anxiety levels. However, some young children may be frightened by clowns and become tearful or anxious. The clown should be trained to notice and understand these children, and staff and parents should be consulted to help reduce anxiety until the particular child is satisfied with the clown. This is consistent with the results of this study. The purpose of article 2 (https://doi.org/10.3389%2Ffped.2024.1324283) was to evaluate the effectiveness of medical clowns in reducing pain and anxiety in hospitalized pediatric patients and their parents in different areas of medicine. The findings suggest that medical clowns have substantial positive and beneficial effects on reducing stress and anxiety in pediatric children and their families in a variety of settings, consistent with the findings of this study.

However, different from these two articles, the purpose of this study is to summarize the timing, form, and content of intervention, outcome indicators, and effects of clown care in hospitalized children. The first two articles only analyzed the application effect of clown care on hospitalized children but did not analyze the intervention timing, intervention form and content, and outcome indicators of clown care in different diseases. At the same time, the pain assessment in the above two articles may be limited to the pain assessment scale based on facial expression and behavior due to the incomplete literature included, while this study noted through literature analysis that the pain assessment tools based on facial expression and behavior may be limited to the measurement of subcortical body and autonomic motor pathways. This does not objectively diagnose the pain experience of the child. Cortisol is the main hormone in the body under stress, which can effectively detect pain in children , the research objects included in the first two articles are mostly children receiving invasive medical operations and surgeries, and this study also includes children with autism and children with cancer, so the research objects are more abundant, and the scope of clown care is wider.

In short, other articles on clown care mostly focused on analyzing the clinical application effect of clown care in hospitalized children. However, compared with other similar articles on clown care, this study not only analyzed the application effect of clown care but also fully analyzed and summarized its intervention timing, intervention form and content, and outcome indicators. In order to provide a reference for medical personnel to carry out relevant intervention.We also present the results of the analysis in the manuscript. See the manuscript for details.

**Reviewer 2:**

Regarding Reviewer 2's comments, we would like to thank the reviewer for taking the time to read our paper and for giving us the opportunity to revise it with many meaningful comments. We have carefully read Reviewer 2's comments and revised each of them in order to take this opportunity to improve the rigor and scientific quality of our paper, as shown in the following responses:

1. Please rephrase this as PLOS Guidelines using the format "Department, Institution, City, State, Country". Adjust font, line spacing and other formatting:

We've rephrased it as the PLOS Guidelines as "Department, Agency, City, State, country,"As follows: School of Nursing, Yunnan University of Chinese Medicine, Kunming, Yunnan, China; Pediatric, Yan'an Hospital Affiliated To Kunming Medical University, Kunming, Yunnan, China.

At the same time, we also reset the font, line spacing, and other formats in strict accordance with the reviewer's annotations and PLOS ONE requirements.

2. ABSTRACT：Please attempt to shorten abstract by following the PLOS guidelines:

Thanks to the reviewer's reminder, We have shortened the abstracts in strict accordance with the requirements of the PLOS guidelines.Please refer to the "Manuscript with Track Changes" and "Manuscript."

3. ABSTRACT:（“Research on the efficacy of clown care for hospitalized children has produced varying results, with differences in treatment duration and methods.” ）This is an important point, but it lacks specificity. What kind of variability in the results has been observed (e.g., effectiveness across age groups or types of conditions)? Clarifying this would help the reader understand the motivation behind conducting a scoping review.

We sincerely appreciate the suggestions provided by the reviewer and have implemented changes based on their feedback. However, due to the constraints of the abstract, we will relocate the enhanced specific content to the introduction section, where it will be more detailed. Please refer to the "Manuscript with Track Changes" and "Manuscript."

4. ABSTRACT：(“A total of 16 articles were included”)The number of articles included in the review is mentioned, but there is no indication of the total number of articles screened or excluded. Including these numbers would provide a better sense of the comprehensiveness of the review process. For example: "Out of X articles screened, 16 were included.":

Thanks to the reviewers' comments, We've updated it to reflect the reviewers' feedback and now read, "Out of 1084 articles screened, 18were included."

5. ABSTRACT：(“Clown care can be used with children who have burns, perioperative care, fracture rehabilitation, autism, puncture therapy, and chemotherapy, according to a review of the literature.”) This sentence effectively summarizes the range of conditions where clown care is applied. However, the results could be enhanced by specifying which conditions showed the most positive outcomes or where clown care was less effective. Additionally, it would be helpful to include specific examples of studies that highlight these applications:

We much appreciate the reviewer's input, and we have made the necessary revisions.Through the presentation of particular research instances for various applications, the most advantageous outcomes of clown care are emphasized. To guarantee that the outcomes are accurate. Please refer to the "Manuscript with Track Changes" and "Manuscript."

6. ABSTRACT：(“Physical, psychological, cognitive-behavioral, and other indications are the several types of clown care outcome indicators”)The sentence is a bit vague. It would be better to briefly explain the specific outcome measures used in the studies (e.g., reduced anxiety, improved coping skills, quicker recovery) and how these were assessed. This would help clarify the effectiveness of clown care interventions across different domains:

Thank you very much for the reviewer's suggestion. We have changed it to "physical (reduced children's pain levels), psychological (reduced anxiety, improved coping skills, quicker recovery), cognitive-behavioral (for example, the number of meaningful words that children with autism say in 10 minutes), and other indications are the several types of clown care outcome indicators". Please refer to the "Manuscript with Track Changes" and "Manuscript."

7. ABSTRACT：(“The study concludes that hospitalized children can benefit from clown care interventions, which exhibit diversity but lack specificity”)This conclusion is reasonable, but it would be stronger if more specifics were provided. What types of interventions or patient populations benefit the most? The phrase “lack specificity” could be expanded—does it mean there’s a need for standardization in delivery methods, or variability in patient responses:

We are grateful to the reviewer for giving us these helpful recommendations. The content has been updated based on your recommendations. "lack of specificity" is elaborated upon, adding more information to strengthen the argument.Please refer to the "Manuscript with Track Changes" and "Manuscript."

8. ABSTRACT：(“Keywords: ”)Write this section please:

Thanks to the reviewer's reminder, I'm terribly sorry. It's our fault. We've added to the keyword section, details as follows: "Keywords: Clown care; Hospitalized children; Scope review; nurse".

9. INTRODUCTION：(“Hospitalization can have adverse effects on an individual's emotional, behavioral, cognitive, and educational development, particularly in children”) Consider elaborating on the specific adverse effects mentioned. Providing examples or referencing studies that quantify these impacts would strengthen the argument:

I sincerely appreciate the feedback from the reviewer. To effectively quantify these negative impacts, we have included targeted references to bolster our position. Additionally, we have outlined the specific adverse effects,the modified content is as follows :

"Hospitalization can have adverse effects on an individual's emotional, behavioral, cognitive, and educational development, particularly in children[1,2,3,4,], due to their limited cognitive abilities and unfamiliarity with the hospital environment.

The child's brain and nervous system remain underdeveloped at a young age. The absence of a clear purpose, coupled with the distress stemming from the illness, proves to be intolerable. The child's surrounding environment is exceedingly sensitive, leading to feelings of fear when confronted with the sight of medical staff in white gowns. Additionally, prolonged exposure to an unfamiliar setting, along with continuous diagnostic and nursing interventions, gives rise to anxiety, depression, and other detrimental emotions. These feelings are expressed through behaviors such as crying and resistance, which negatively impact the child's willingness to comply with medical directives and hinder the effective progression of clinical diagnosis and treatment."

10. INTRODUCTION：(“causing significant stress reactions”)The use of "significant stress reactions" could be clarified. It may be beneficial to specify types of stress reactions (e.g., withdrawal, aggression) to illustrate the point more effectively:

I sincerely appreciate the feedback provided by the reviewers. We have clarified the usage of "major stress response" in the manuscript. At the same time, the specific types of stress response are also explained. Certain "significant stress reactions" that can manifest in hospitalized children are thoroughly clarified by discussing specific stress-related behaviors (such as crying and resisting medical interventions, among others).Please refer to the "Manuscript with Track Changes" and "Manuscript."

11. INTRODUCTION:(“Therefore, it is crucial to assist hospitalized children in acclimating to their new surroundings and implementing effective interventions to alleviate their concerns and anxieties”)You may consider briefly mentioning other interventions currently employed for hospitalized children to highlight the necessity for clown care as a complementary or alternative approach:

We are very grateful to the reviewers for their suggestions. Based on your suggestions, we have revised the original sentence and added other interventions currently used for hospitalized children to emphasize the need for clown care as a complementary or alternative approach. Please refer to the "Manuscript with Track Changes" and "Manuscript."

12. INTRODUCTION:(“Clown care, also known as clown therapy or medical clowning, has emerged as a popular non-pharmacological intervention in pediatric care, geriatrics, and chronic disease management in recent years”)If the primary focus of the review is pediatric care, clarify this early in the introduction. Mentioning geriatrics and chronic disease management may divert focus unless these populations are also addressed in the review:

We sincerely appreciate and concur with the reviewer's insights. In alignment with the reviewer's feedback, we have removed references to geriatrics and chronic diseases from the manuscript to clarify that the primary focus of this study is on hospitalized pediatric patients.

13. INTRODUCTION:(“Rooted in humor”)Consider defining what constitutes "humor" in the context of clown care. Adding a few examples of specific techniques:

We are very grateful to the reviewers for their suggestions. This is an excellent recommendation. If it is presented solely in a humorous context, some individuals may not grasp the specific implications. We have incorporated explanations based on your recommendations to enhance clarity, and the modified content is as follows:

"Clown care is a non-drug intervention method based on humor. It is mainly manifested in the following four aspects.For instance, from a physiological perspective, patients can release endorphins through humor and hearty laughter, thereby regulating and stimulating their immune response. Cognitively, clowns embody various roles that divert patients' attention from their conditions, fostering imagination and facilitating self-expression. In terms of emotional well-being, patients are encouraged to substitute negative feelings with positive ones. Socially, clowns engage with patients to enhance interaction and connection. It is upon this foundation of humor theory that clown care is recognized for its valuable contributions within medical environments."

14. INTRODUCTION:(“Clown care programs have been widely adopted internationally since the emergence of hospital clowns in North America in the 1980s; currently, clown doctor initiatives are available in various regions including the US, Canada, Europe, Israel, Australia, South Africa, Asia, and South America ”)Consider adding context on the effectiveness or impact of these programs internationally. Mentioning notable outcomes or evaluations could support the argument for their relevance:

I sincerely appreciate the reviewer's recommendations. In line with your suggestions, we have reviewed pertinent literature and enhanced the section on the historical development and international influence of clown care. The revisions are as follows:

"In the early 20th century, two clowns first appeared in a pediatric ward in London. In the 1980s, Michael Christensen, a professional clown, founded the Clown Care Center in New York. In the same year, Canadian child life expert Karen Ridd established the Clown Care Program at Winnipeg Hospital. Since then, clown care has been formally integrated into the medical and nursing process. Hospital clowning continues to grow around the world. Numerous hospitals across the United States, Canada, Europe, Israel, Australia, South Africa, Asia, and South America presently implement clown intervention programs."

15. INTRODUCTION:(“primarily due to cultural differences”)It would be beneficial to elaborate on the specific cultural differences that hinder the development of clown care in China:

Thank you very much for the feedback provided by the reviewers. We have incorporated your observations regarding the specific cultural differences that pose challenges to clown care. In Western contexts, clowns typically originate from circus performances, captivating audiences with theatrical acts (often in a pantomime style) and their flamboyant, vividly colored costumes. In contrast, Chinese children have limited exposure to clowns as a concept; they are more familiar with traditional Chinese animated figures such as Sun Wukong and Zhu Bajie from "Journey to the West." Consequently, due to these cultural distinctions, the conventional red-nosed clown in clown care may be more difficult for Chinese children to accept and may even induce fear.

16. INTRODUCTION:(“Variations in staff qualifications, intervention protocols, and research methodologies exist, with limited relevant studies conducted in China.”)You may want to specify how these variations could affect the outcomes of clown care interventions. Adding a statement about the need for standardization in protocols could also enhance the discussion:

We sincerely appreciate the reviewer's suggestions. In response to your suggestions, how do these differences affect the effectiveness of interventions in clown care. The detailed changes are outlined below:

"In contrast to other countries, the implementation of therapeutic clowning for hospitalized children in China has been limited in duration. The majority of "clown doctors" are volunteers, which results in a lack of an organized training framework. Additionally, there is no standardized procedure regarding the recruitment criteria, sources, conditions, training methodologies, content, or length of training for "clown doctors."

17. INTRODUCTION:(“Literature from both domestic and international sources was reviewed and analyzed to outline the structure and content of clown care interventions, the optimal timing for interventions in hospitalized children, as well as the outcome indicators and effects.”)Consider rephrasing for conciseness: "Literature from domestic and international sources was reviewed to outline the structure, content, optimal timing, and outcome indicators of clown care interventions for hospitalized children." This makes the sentence clearer and more direct:

We sincerely appreciate the insights and corrections provided by the reviewer. We have revised the original sentence in line with the reviewer's recommendations.Please refer to the "Manuscript with Track Changes" and "Manuscript."

18. METHODS:(The measures used by clown care in hospitalized children are as follows?)Rephrase this statement into a clear question or remove it if it does not lead to a concrete research question. It seems incomplete as a standalone statement:

I sincerely appreciate the reviewer's recommendations. After thorough discussions, we recognized that it was unlikely to yield a definitive research question. Upon careful reflection on the reviewer's feedback, we ultimately resolved to remove it from the original manuscript.

19. METHODS:(“To address discrepancies, examiners revisit inclusion and exclusion criteria and perform additional screening.”)You might clarify how discrepancies were resolved if they arose. Was consensus reached through discussion or additional criteria?

I sincerely appreciate the recommendations provided by the reviewers. Based on your suggestions, we have re-evaluated our approach to address the discrepancies and have made the necessary revisions. Please refer to the "Manuscript with Track Changes" and "Manuscript."

20.  (“Once the data were plotted, a third researcher checked the data's quality and made any necessary updates to the extracted data to include all the data and improve the study's overall completion.”)Clarify what you mean by "all the data." Additionally, consider emphasizing how the data quality was assessed to reinforce rigor.

We greatly appreciate your professional comments on our articles.We would like to clarify the term "all data," which specifically refers to the thorough extraction of all relevant information from the collected literature. This includes the following components:① title, ② author, ③ nation, ④ publication date, ⑤ kind of study, ⑥ sample size, ⑦study population , ⑧ intervention strategy, ⑨ outcome indicators. To elaborate, after two researchers extract the data, a third researcher reviews it to confirm that all the specified details have been included. We apologize for any ambiguity regarding our terminology as it may have lacked precision. Additionally, in response to your feedback, we have added a section on assessing the quality of the data to improve its rigor. The revised sentence is as follows:"Once the data were plotted, the third researcher, through repeated reading and analysis of the included literature, checked the extracted data and results, and updated the extracted data as needed, so as to obtain all the data to make the study more complete and form the final results."

21. METHODS: (What pediatric patients of clown care are provided to children in hospitals?)This question is somewhat unclear. Consider rephrasing to specify what is meant by "what pediatric patients of clown care." It may benefit from restructuring to clarify its intent.

We sincerely appreciate the reviewer for bringing this issue to our attention. Due to our oversight, we failed to provide an accurate translation of the sentence, which led to ambiguity in its meaning,we are very sorry. We promptly addressed this matter and retranslated it. The intended meaning of the sentence is primarily:“What types of illnesses does clown care predominantly address in pediatric inpatients?”

22. METHODS:(“In what ways and with what pediatric patients of interventions does clown care get applied to pediatric patients in hospitals?”) This question could be more direct. Rephrasing for simplicity and clarity, e.g., "How is clown care applied in pediatric settings, and which patients benefit most?

I greatly appreciate the reviewer's insightful suggestions. Following your recommendations and our collaborative discussions, we realized that the example you provided effectively clarifies the specific issue in a simpler and clearer manner. Consequently, we have revised our approach to align with your example. Thank you once again for your invaluable assistance.

23. METHODS:(“This will function as a story with pertinent themes. To the extent feasible, figures and tables will also be utilized to present the findings.”)Ensure that the wording around the use of figures and tables is precise. Consider adding that the visual aids will support the narrative and provide clarity to the findings.

Thank you very much for the reviewer's suggestion. The use of charts to show the survey results here means that the collected data is summarized and analyzed by drawing charts. We have shown all the survey results in Table 2 (General characteristics of the included studies).

24. RESULTS:(“After removing 653 duplicate entries from the initial 1082 pieces of literature” )The removal of duplicates is clear, but the inclusion criteria for the remaining 429 articles should be explicitly mentioned. Were these criteria predefined, and were there any limitations noted in this selection process:

Thank you very much for the reviewer's suggestions, which are very helpful to us. We have supplemented the inclusion criteria for the remaining 429 articles in the manuscript. These standards are set pre-emptively. These articles were selected by excluding those that did not fit the content and topic of the study substances, as well as those that cannot be accessed in full text.At least two reviewers screened each result by title and abstract, with disagreements resolved by discussion with the review team. The remaining full text papers were accessed and screened against the eligibility criteria, again by at least two reviewers with disagreements resolved by discussion.

25. RESULTS:（“Among these, 11 were in English and 5 were in Chinese.”）While the inclusion of studies from different languages is commendable, how were non-English studies handled? Was any translation or verification process employed? Were there any potential biases due to language limitations:

Thank you very much for the reviewer's suggestions, and we also recognize that there may be potential translation bias in the Chinese study. However, Chinese is our mother tongue, so we used professional translation software to translate it into English, strictly following the basic requirements for translation of different languages. At the same time, in order to ensure the correctness and scientificity of the translated language, we also consulted an English expert for revision.We are confident that our scientific and meticulous translation methodology eliminates any risk of bias stemming from language discrepancies.

26. RESULTS:(“Evaluation of clown care often involves measuring parent satisfaction.”)The mention of parent satisfaction is important, but it would be useful to detail how satisfaction was measured (e.g., specific surveys, Likert scales) and discuss the reliability or limitations of these subjective evaluations:

I sincerely appreciate the valuable feedback offered by the reviewer. In light of your recommendations, we have made adjustments and included a methodology for assessing the satisfaction of parents with hospitalized children, as well as a discussion of the specific reliability and limitations. The revised statement is as follows:

"The evaluation of caregiver satisfaction primarily involved the use of questionnaire surveys or follow-up assessments of healthcare personnel. This methodology allows for a swift and straightforward capture of the subjective experiences reported by the parents of hospitalized children, making it both practical and accessible. However, it is important to note that this approach may lack the precision and objectivity associated with standardized assessment scales."

27. RESULTS:The qualifications of clown practitioners should be discussed in greater detail. Were the practitioners standardized in terms of training or background, and how might practitioner variability affect the consistency of outcomes?

We sincerely appreciate the reviewers' insightful advice, which was greatly appreciated. We have examined the pertinent literature in light of your recommendations and added an explanation of the requirements for clown care practitioners' eligibility as well as how variations across practitioners impact the consistency of outcomes. Clown doctors do not have a set criterion for qualification.Only the basic rules of public morality were laid down. Clown doctors are not subject to uniform standards regarding their recruitment, sources, circumstances, training techniques, content, or duration. The application effect of clown care may be somewhat impacted by the diversity of practitioners. In the future, there should be a unified protocol for the participation of clown physicians. Please refer to the "Manuscript with Track Changes" and "Manuscript."

28. RESULTS:(The elements of clown care can be encapsulated by terms such as "cognitive coping,")A good introduction, but practical examples of how cognitive coping was facilitated in these studies would strengthen the reader’s understanding of its real-world application.

I greatly appreciate the reviewer's recommendations. To enhance readers' comprehension of the nuanced meaning of cognitive coping, we have incorporated practical examples of cognitive coping in accordance with the reviewer's guidance. This addition ensures that readers grasp the specific definition of cognitive coping. The revised content is detailed as follows:

“Cognitive coping is designed to encourage pediatric patients to actively respond to the challenges brought by the disease and improve their self-cognitive ability. Studies have shown that "medical clowns" improve their self-cognitive ability by guiding perioperative pediatric patients to "Give yourself a like" other positive behaviors to improve their self-cognition ability.”

29. RESULTS:("Distraction"[10,11,12,13,14,15,16,17,18,19,20,21,22,23]involves medical clowns dressing up as beloved characters”)While distraction is well-explained, further analysis on whether certain types of distractions (e.g., storytelling vs. physical comedy) were more effective, especially across different age groups, would be valuable.

Thank you very much for the reviewer's suggestion, which is a very valuable suggestion. Based on your suggestions, we have analyzed that providing personalized entertainment programs for different age groups of children may have a more noticeable effect on distracting attention. The specific modifications are as follows:

"Distraction" pertains to the use of medical clowns who engage with children through enjoyable experiences designed to capture their attention. These interactions can be tailored to the child's age and may include magic tricks, pantomime, storytelling, and music, among other forms of entertainment. For instance, adolescents might appreciate magic performances or juggling acts, while younger children may enjoy fairy tales or their favorite children's songs. By providing customized entertainment programs aligned with the age and interests of hospitalized children, we can more effectively alleviate discomfort and anxiety during their stay, thereby diverting their attention from distressing circumstances.

30. RESULTS:(“Psychological markers were reported in seven studies”)A deeper analysis is needed here to clarify whether clown care consistently demonstrated superior psychological outcomes compared to standard care or other non-pharmacological interventions.

We are very grateful to the reviewer for providing us with such helpful suggestions.We re-read and analyzed 7 studies that included psychological indicators, and the final results all showed that the psychological intervention results of clown care were always better than standard care and other non-drug interventions.The specific modifications are as follows:

"This study's seven included studies demonstrated that, when compared to standard care, children and adolescents who had hospital clowns—whether or not a parent was present at the time of intervention—reported much less anxiety and improved psychological adjustment."

31. DISCUSSION: (“Clown phobia refers to the extreme aversion and distress experienced by individuals towards clown figures. Each patient is a unique individual with distinct characteristics.”)This is a valid point, but elaborating on what specific characteristics need to be considered (age, cognitive ability, cultural background, etc.) would add clarity:

We appreciate your advice and find it extremely helpful. Based on your recommendation, In order to identify the precise traits (age, cognitive capacity, cultural background, etc.) that should be taken into account for clownphobia, we searched the literature.It is also explained in the manuscript.Please refer to the "Manuscript with Track Changes" and "Manuscript."

32. DISCUSSION:(“The onset of these phobic reactions in children was found to be unrelated to factors such as race, religion, specific illnesses, or associated symptoms.”) The finding is interesting, but it would be useful to explain why these factors were initially thought to influence clown phobia, and why they ultimately did not.

First of all, we are very grateful to the reviewers for their scientific rigor, and we are very sorry for this, as our lack of rigor and conscientiousness has caused misunderstanding to the reviewers.When the reviewer raised the issue, we immediately went back and reviewed the original text. We found that we had misunderstood the meaning of the original text due to a translation error. We looked for qualified English experts to help us properly translate the quoted material. What this study really means is that there is a correlation between race, religion, disease, and the symptoms and indicators of clownphobia in children.We apologize again for this.

33. DISCUSSION:（“research indicates that symptoms of clown phobia can persist for up to 60 years”) This long-term effect is concerning. More explanation on the psychological mechanisms behind such persistent phobias would enhance the reader’s understanding of the potential risks.

We strongly agree with the reviewer's suggestions. Therefore, in accordance with the reviewer's suggestions, we have explained the psychological mechanism behind clown phobia in detail as follows:

"Theories of the aetiology of clown fear can be broadly split into three general categories: those relating to their physical appearance, those relating to their behaviour and those derived from learning and/or experience. Firstly, considering fear of clowns as deriving from aspects of physical appearance,  suggests that coulrophobia may stem from the uncanny valley effect which describes the feelings of eeriness and repulsion triggered by near human-looking objects.With regard to clowns, the distortion of their facial features through makeup gives them a ‘near-human’ quality which may elicit the uncanny valley effect.Secondly, behavioural explanations of clown fear focus on the unpredictability of their behaviour.Clowns laughing at random intervals, as well as the nature of their performances emphasizing magic and trickery, may make the child feel uncomfortable or upset.Finally, the fear of clowning may stem from the negative portrayal of clowns in the media, as well as from negative experiences in the same year."

34. DISCUSSION:(“Furthermore, 89.5% of patients reported no significant improvement in their fear of clowns, nor did it alleviate social or psychiatric issues.”)This is a striking statistic. Was this result specific to a certain type of clown care intervention? Clarifying the intervention type could provide more insight into why these outcomes were observed.

# First of all, we are very grateful to the reviewers for their scientific rigor, and we are very sorry for the misunderstanding caused by our lack of rigor. Upon discovering this issue, we immediately checked the data and did not find it in this article. After realizing the seriousness of the problem, we immediately reread and analyzed the literature, and invited professional English experts to help us translate and find the cause of the error. The actual figure was 8.9%, but due to our mistakes and careless interpretation of the data, it was accidentally revealed to be 89.5%. In the manuscript, we also specify that this data originates from a study on the clinical characteristics of clownphobia. I would like to extend my apologies once more. We will ensure to take this error to heart and prevent similar oversights in the future.

35. DISCUSSION:(“To effectively manage and alleviate the pain experienced by hospitalized children, it is essential to conduct a thorough and precise evaluation of their discomfort.”)This is a strong assertion, though it could benefit from mentioning some established pain assessment methods commonly used in pediatrics as a baseline for comparison.

We strongly concur with the reviewer's recommendation and its implications. Consequently, we have incorporated several widely recognized pain assessment techniques for children at the conclusion of this paragraph to provide a foundational reference for future precise assessment methodologies. Please refer to the "Manuscript with Track Changes" and "Manuscript."

36. DISCUSSION:(“Cortisol, the primary stress hormone produced by the body, has emerged as a valuable objective marker for evaluating children's discomfort and the effectiveness of treatments .”)The inclusion of cortisol is a solid argument, though the discussion could be improved by mentioning how cortisol levels might fluctuate due to factors unrelated to pain (e.g., time of day, other stressors).

We wholeheartedly concur with the recommendations made by the reviewer, which have proven extremely beneficial to our work. In line with the reviewer's advice, we promptly reviewed pertinent literature and discovered that cortisol levels exhibit a circadian rhythm fluctuation. Clinical practitioners may select optimal sampling times based on the natural timing of cortisol's circadian variation. The specific supplementary content is:

"When the body encounters pain stimuli, it elicits a corresponding neuroendocrine stress response, leading to an elevation in cortisol levels. Morelius et al. [68] demonstrated that painful interventions, such as fundus examinations and heel blood draws, can elevate cortisol levels in neonates, while certain non-pharmacological interventions may mitigate these cortisol levels. Cortisol secretion exhibits a circadian rhythm. Human cortisol metabolism adheres to a 24-hour physiological cycle, peaking between 6:00 and 8:00 AM, followed by a gradual decline, with the lowest levels occurring from midnight to 2:00 AM the subsequent day. Ivars et al. [72-73] undertook a 12-month study measuring salivary cortisol in healthy full-term and preterm infants, revealing that the salivary cortisol rhythm is established during the neonatal phase (one month postpartum), characterized by higher levels in the morning compared to the evening, which then stabilize over time. To mitigate the effects of circadian rhythm, clinical sampling should be conducted at the time of maximum cortisol concentration."

37. DISCUSSION:(“Among the 16 studies included in this investigation, only one [23] utilized salivary cortisol as an outcome measure to assess pain in children.”) This highlights a gap in the literature, but the paper would benefit from discussing why this marker has been underutilized and whether there are practical barriers to implementing cortisol testing in clinical settings.

We extend our sincere appreciation to the reviewers for their insightful feedback on our manuscript. In response to your recommendations, we have elaborated on the rationale behind cortisol being an underexploited biomarker. Typically, cortisol is employed to evaluate pain intensity during invasive procedures, whereas its application in non-pharmacological strategies to alleviate pain remains infrequent. This limited utilization can be attributed to the intricate nature of the body's endocrine system, wherein various hormones may interact with one another. The specifics are outlined below:

"Currently, the utilization of salivary cortisol for assessing neonatal pain predominantly centers on planned pain responses. There is a scarcity of research investigating variations in salivary cortisol levels in newborns following non-pharmacological therapies. This gap is largely attributed to the intricate nature of endocrine dynamics, which may have interplay among different hormones. Research conducted by Pavlyshyn et al. indicates that interventions such as skin-to-skin contact can elevate oxytocin levels while concurrently diminishing salivary cortisol levels, thus enhancing stress responses in preterm infants. Both endorphins and oxytocin possess analgesic and pleasurable effects and serve as anti-stress hormones, whereas cortisol is regarded as the primary hormone associated with stress. Further research is necessary to delve deeper into the interactions between hormones, pain, and their underlying mechanisms."

38. CONCLUSION:(“Children in hospital settings experience significant benefits from clown care interventions that positively impact their physical, mental, and cognitive well-being.”)This is a strong statement. However, adding more specific examples from the findings (e.g., reductions in anxiety or pain) would provide concrete evidence to support this claim.

I sincerely appreciate the valuable feedback provided by the reviewer. In response to your recommendations, we have incorporated additional specific examples, such as the alleviation of anxiety or pain, derived from the study's findings to strengthen the validity of the evidence. The detailed supplementary content is outlined below:

"The application of therapeutic clowning can divert pediatric patients' attention from illness and discomfort, alleviate their pain, mitigate their anxiety and tension, and enhance their adherence to medical treatment protocols as well as their overall satisfaction with healthcare services."

39. (“The results highlighted the favorable and successful effects of implementing clown care for children in hospital settings”)This is a valid point, though it would be stronger with more specifics about which aspects of the interventions were most successful (e.g., emotional reflection, distraction).

We strongly agree with the reviewer's suggestions, and we are also very grateful to the reviewer for their valuable suggestions. Based on your suggestions, we have added where clown care has been successful to ensure the evidence is stronger. The specific supplementary content is as follows:

For instance, regarding the “Guided imagery”, pediatric patients are prompted to engage actively with the challenges posed by their illness to enhance their self-awareness. In terms of empowerment, the therapeutic clown adapts their actions based on the pediatric patient's "guidance," allowing the patient to experience a sense of autonomy and boost their self-esteem.“Divert attention”, pediatric patients are encouraged to manage painful experiences by envisioning a cherished object or recalling a pleasurable memory to redirect their focus.

40. (“Additionally, the utilization of physiological markers such as salivary cortisol was infrequent, with measures and questionnaires being the predominant assessment tools for evaluating the impact of clown care”)This is a solid observation. Adding a sentence on why physiological markers like cortisol could be a valuable addition to the existing methods would strengthen the argument for their inclusion.

Thank you very much for the reviewer's suggestion. We also think it is very valuable to add that sentence, and we have added that sentence according to your suggestion. The specific supplementary contents are as follows:

"In comparison to the observational evaluation of behaviors like facial expressions and body language, cortisol serves as the primary hormone indicative of stress in the body. Its collection method is straightforward and non-invasive, which can make the detection results more accurate."

41. (“To establish a robust and scientifically grounded framework for the clinical advancement of high-quality clown care programs for hospitalized children, future research should explore the influence of various cultural backgrounds and values.”)Great recommendation, though it could benefit from being more specific about how cultural differences might shape clown care interventions. For instance, which cultural factors should be prioritized, and how might they affect both the perception and effectiveness of these programs?

We strongly agree with the reviewer's suggestions,which have been extremely beneficial to our work. In response to your feedback, we have also incorporated which cultural factors should be emphasized and their impact on the perception and efficacy of these programs. The detailed supplementary content is outlined below:

"Given the cultural disparities between China and Western nations, it is essential for Chinese medical institutions to develop an optimal clown care implementation strategy for pediatric patients in China. This should be informed by insights gained from international clown care practices while being tailored to align with the unique social and cultural context of China. Firstly, elements such as magic and other engaging content that resonate with Chinese children should be preserved. Conversely, incorporating Chinese cultural motifs into the "clown doctor" persona—such as substituting the Western "red nose" clown with traditional Chinese cartoon characters—can enhance its appeal to pediatric patients."

Once again, I would like to thank the editor and reviewers for their comments and giving me the opportunity to revise my paper. Through this thesis revision process, I deeply realized the shortcomings in our research and made comprehensive and in-depth improvements under your guidance. I believe that after these revisions, the quality of the thesis has been significantly improved and is more in line with the publication standards of PLOS ONE journals. Once again, I would like to thank you for your hard work and valuable comments and look forward to your further review and affirmation.
