# Supplementary material for: Application of clown care in hospitalized children: A scoping review
Source: PLoS One. 2024 Dec 19;19(12):e0313841. doi: 10.1371/journal.pone.0313841 (PMC11658477; doi:10.1371/journal.pone.0313841)
Supplement: Attachment — Submitted filename: Response to Reviewers.docx [file pone.0313841.s007.docx]

**Response to Reviewers**

Dear Editors:

Greetings!

First of all, on behalf of all the authors, please allow me to extend our sincerest thanks to you and the reviewers. We would like to thank you for taking the time out of your busy schedules to review our paper in detail and provide valuable comments and suggestions. We are fully aware of the importance of these comments in improving the quality of the paper and ensuring academic rigor.

After receiving the review comments, we immediately organized our team to conduct in-depth discussion and analysis, and revised and improved the paper in strict accordance with the editors' suggestions. Editorial comments are listed below in red font. such as below, where specific questions are numbered. Our responses are given in black font. The following are our responses and explanations to the editorss' comments:

1. Please review your reference list to ensure that it is complete and correct. If you have cited papers that have been retracted, please include the rationale for doing so in the manuscript text, or remove these references and replace them with relevant current references. Any changes to the reference list should be mentioned in the rebuttal letter that accompanies your revised manuscript. If you need to cite a retracted article, indicate the article’s retracted status in the References list and also include a citation and full reference for the retraction notice:

I sincerely appreciate your guidance. At the same time, we sincerely apologize for any inconvenience our errors may have caused you. After receiving your recommendation, we looked over the references right away and, per your request, deleted the ones that had been withdrawn and substituted the most recent ones. Again, I apologize. Please refer to the "Manuscript with Track Changes" and "Manuscript."

2. Thank you for your revision. Please shorten your conclusion and place it under the heading "Implications.":

I appreciate your suggestion very lot. In response to your suggestion, we have condensed the conclusion and placed it under "Implication".Please refer to the "Manuscript with Track Changes" and "Manuscript."

Once again, I would like to thank the editor and reviewers for their comments and giving me the opportunity to revise my paper. Through this thesis revision process, I deeply realized the shortcomings in our research and made comprehensive and in-depth improvements under your guidance. I believe that after these revisions, the quality of the thesis has been significantly improved and is more in line with the publication standards of PLOS ONE journals. Once again, I would like to thank you for your hard work and valuable comments and look forward to your further review and affirmation.
